# Spontaneous Ogilvie's Syndrome in a Nonagenarian Male: A Study of a Rare Case With Literature Review

**DOI:** 10.7759/cureus.30992

**Published:** 2022-11-01

**Authors:** Fahad Hussain, Abdul Waheed, Mian Hamza Hamdullah, Farah Ismail, Haseeb Mehmood Qadri, Nauman Khurshid

**Affiliations:** 1 General Surgery, Lahore General Hospital, Lahore, PAK; 2 General Surgery, Mayo Hospital, Lahore, PAK; 3 General Surgery, University of Lahore Teaching Hospital, Lahore, PAK

**Keywords:** cute colonic pseudo-obstruction, laparotomy, colonic decompression, neostigmine, ogilvie’s syndrome

## Abstract

Ogilvie's syndrome, or acute colonic pseudo-obstruction (ACPO), is an occasional disorder that occurs in hospitalized patients who have undergone major surgery. It presents with the clinical features of intestinal obstruction without any definitive intrinsic or extrinsic anatomical cause. Without prompt treatment, it can lead to life-threatening complications. The risk factors of this syndrome must be sought to prevent its occurrence. We report a rare case of idiopathic, spontaneous, and non-traumatic Ogilvie's syndrome, with old age as the only present risk factor in our case. To our best knowledge, this is the first-ever case reported in English scientific literature from Pakistan.

## Introduction

Acute colonic pseudo-obstruction (ACPO) is a rare functional and structural gut motility disorder resembling mechanical obstruction [1]. It was first described by Heneage Ogilvie in 1948 [2]. Worldwide, it has an incidence of 60% in males, and the mean age of its occurrence is the sixth decade of life [3]. Predisposing factors to this syndrome include non-operative trauma, infection, cardiac disease, and neurological disease, along with cardiac and orthopedic surgery [4]. Disturbance of autonomic innervation, usage of neurotropic medications, and metabolic disorders (commonly hypokalemia and uremia) are some notable pathophysiologic mechanisms involved in its causation [5]. It is also thought to be associated with diabetes, hypothyroidism, and some autoimmune disorders [5]. Clinical features include abdominal pain and distension, vomiting, constipation, and diarrhea [3,6]. A plain abdominal radiograph is the first-line screening and diagnostic investigation. But computerized tomography (CT) abdomen is the gold standard investigation as it delineates the intraluminal, intramural, and extraluminal causes of mechanical obstruction remarkably [1,3,6,7]. Its management includes conservative measures, pharmacologic therapy with intravenous neostigmine, and colonic decompression, with surgery being the most definitive and last resort option, including colonic resections and colostomies [2,3,4,5,7,8].

## Case presentation

**Figure 1:**
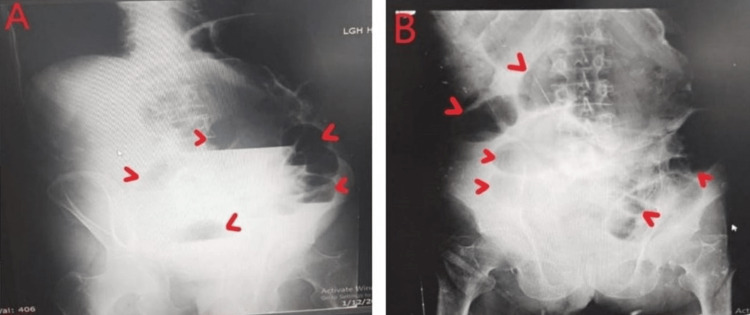
Dilated large gut can be seen with multiple air fluid levels at presentation (A); after 12 hours, the unresolved features can be appreciated (B)

A 90-year-old man with a body mass index (BMI) of 26.9 kg/m^2^ was referred from District Headquarter Hospital, Kasur, Pakistan, to our tertiary care set up, Lahore General Hospital, Lahore, Pakistan, with the reason of referral of "abdominal distension of unknown cause" for the past three days in January 2022. There were failed attempts of abdominal decompression in the parent hospital; hence the patient was referred. His past medical history was significant for non-compliant hypertension and smoking, but insignificant for any medications or family history. He presented with absolute constipation and multiple episodes of nausea and vomiting for the last three days. He had no past medical or surgical history of significance. On examination, his abdomen was non-tender and distended with generalized dull pain. Bowel sounds were hyper-tympanic on auscultation with a hyper-resonant note on percussion. The rest of the clinical examination for other systems was unremarkable. A note of 450ml fecal matter was made in his nasogastric (NG) tube. Chest X-ray (CXR) was normal. The electrocardiography (ECG) of the patient revealed mildly tall and tented T waves in leads V1, V2, and V3, suggesting hyperkalemia. However, the laboratory tests on the day of presentation yielded all baseline blood and serum tests within the physiological range. A digital rectal examination (DRE) showed no fecal staining. Ultrasound abdomen and pelvis (US) showed sluggish bowel movements and a cecal diameter greater than 11 centimeters. X-rays of the abdomen, erect and supine, were performed within the first hour of presentation, revealing a distended large gut with some air-fluid levels (Figure 1). The patient was admitted on the line of sudden and spontaneous colonic obstruction of unknown cause. Initially, the patient was provided conservative management as per protocol. The findings on the X-ray abdomen, erect and supine, remained the same at the twelfth hour, despite conservative measures (i.e., glycerin suppositories) to relieve constipation. There were no findings evident of obstruction on abdominal computerized tomography (CT) scan without contrast. Since rigid or flexible sigmoidoscopy was not readily available, so definitive cause of obstruction was not established. For further evaluation, the consultant on call decided to explore the patient, owing to the three-day history of obstipation, color and quantity of nasogastric tube aspirate, an abnormally large cecal diameter, and deteriorating hemodynamic parameters.

**Figure 2:**
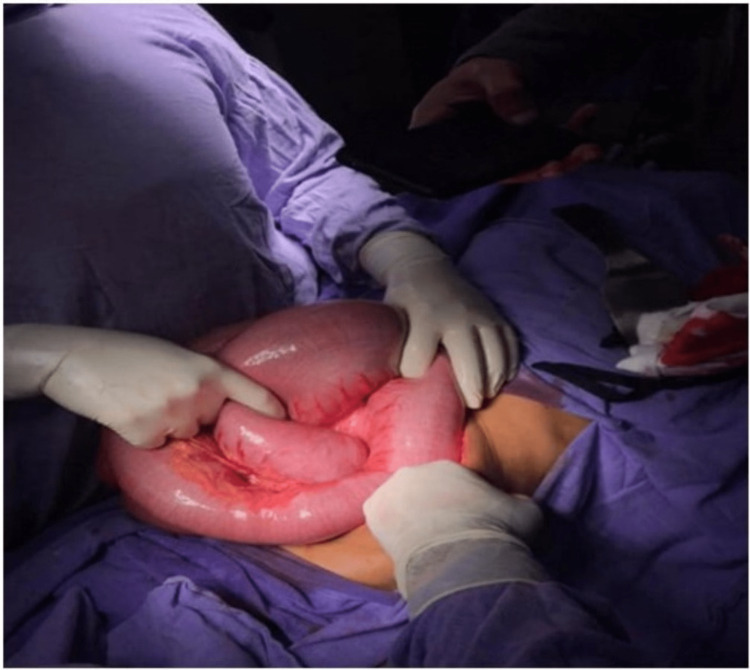
View of intra-operative dilated gut loops in situ

After counseling and taking written consent from the patient and his attendants, the operating surgeons performed a midline, exploratory laparotomy. An abnormally dilated cecum with a diameter of 13cm, along with the whole large gut dilated throughout its length and a long mesentery were the intra-operative findings noted (Figure 2). No adhesions, strictures, or other causes of obstruction could be found. The large gut was decompressed using a 10 cc syringe, and a Faucher's rectal tube was inserted. The patient recovered smoothly from general anesthesia. There were no significant intra-operative or post-operative complications. He was shifted to ward, kept nil per oral, and started on parenteral nutrition. Intramuscular injection of neostigmine 2.5 mg twice a day and per oral tablets of pyridostigmine 60mg twice a day was initiated to start and enhance colonic motility. Serial ECGs and laboratory tests were performed after the addition of supplementary potassium to parenteral fluids. The patient passed flatus after 36 hours of presentation in emergency, and the passage of 100g of stool was documented on the third post-admission day. Due to improvement in his gut mobility after administration of neostigmine and pyridostigmine along with associated senile age, he was diagnosed as a case of senile Ogilvie's syndrome post-operatively. The nasogastric tube was removed the same evening, and the patient started on enteral nutrition with orals slips, shifting to clear fluids, semisolid food, and solid food on the later fourth to sixth post-admission days. He was discharged home later on and followed up in an outpatient clinic every week for a month, biweekly in the second month, and monthly til the sixth month. Throughout these visits, the patient gave a history of normal bowel habits. His midline abdominal wound healed normally. Our patient is living a healthy life nowadays. 

## Discussion

Acute colonic pseudo-obstruction (ACPO) is a term used to characterize a clinical syndrome with symptoms, signs, and radiographic appearance of large bowel obstruction without any mechanical cause [3]. Here we present a unique case of idiopathic, spontaneous, non-traumatic, and non-infectious case of Ogilvie's syndrome. We point out senility to be the likely risk factor in this case study. It is commonly seen after the sixth decade of life, predominantly in males. However, the patient in our study was a male in the ninth decade of his life.

Acute colonic pseudo-obstruction (ACPO) is documented to be caused by dysautonomia, history of surgical intervention, and iatrogenic medications usually [2]. Varicella zoster viral infection has been associated with Ogilvie's syndrome in over 30 cases [9]. In contrast, our patient had no metabolic disorder, i.e., hypokalemia, and history of prior medications and surgical procedures in his life. 

The clinical findings are usually dominated by marked gaseous abdominal dilatation, which is paradoxically well tolerated clinically without deterioration of the patient's condition [2]. The systemic examination in our patient also revealed a distended abdomen with hyper tympanic sounds on auscultation. Plain abdominal radiographs should be the first screening tool showing dilated bowel loops with or without fluid levels [1]. Similarly, erect and supine abdominal X-rays in our patient showed dilated cecum and abnormally distended large gut with some air-fluid levels.

Treatment is usually conservative, including nil per oral regimen, intravenous hydration, discontinuation of narcotics and sedatives, and other precipitating factors [3]. Our approach to managing the patient was analogous in this regard, with discontinuation of any kind of intake, parenteral nutrition, adequate analgesia, and a well-defined antibiotic cover. Neostigmine is the drug of choice in limiting further colonic dysmotility and dilatation as it enhances the parasympathetic action of the autonomic nervous system on the gut [10]. Similarly, the patient in our case report was treated with neostigmine along with potassium supplements. 

Colonic decompression is the initial invasive procedure of choice for patients with marked cecal distension of significant duration (> three to four days), lack of improvement after 24-48 hours of supportive therapy, and those with contraindications or who fail pharmacological therapy (neostigmine) [4]. With no improvement in our patient's clinical condition and a history of greater than three days, exploratory laparotomy with colonic decompression using a 10 cc syringe was performed and a Faucher's rectal tube was also placed. Surgery cecostomy is reserved for very ill patients or those with evidence of colonic ischemia or perforation [3]. Our patient had none of these indications, and cecostomy was not opted for.

Bowel perforation is the most serious complication, and the risk increases substantially if the colon remains distended [10]. General guidelines indicate a cecal diameter >9cm to be abnormal and >12cm to be a strong risk of perforation. The thin-walled cecum is the most common point of perforation [10]. In our case, the patient was discharged after the passage of stool and was closely followed up in outpatient for three months. He lived a healthy life with normal bowel habits and no post-operative complications.

## Conclusions

As seen in this 90-year-old patient, despite his normal investigations along with the absence of a history of medication or surgery, he developed a spontaneous pseudo-obstruction which responded to promotility parasympathomimetic drugs post-operatively. Hence, the primary risk factor appears to be extreme age in this case. Further studies are essentially required to seek out the pathophysiologic mechanisms related to old age, which predisposes to Ogilvie's syndrome spontaneously.

We attempt to provide an insight to clinicians that not all cases of Ogilvie's syndrome can be identified with the usual history of metabolic derangements, medication intake, or post-surgery. We should consider these clinical manifestations and higher extremes of age as a paramount factor in labeling a patient with Ogilvie's syndrome.
